# Research on multiple improvement paths of national innovation output based on tsQCA

**DOI:** 10.1371/journal.pone.0333637

**Published:** 2025-10-10

**Authors:** Zhenxing Gong, Xiangge Wang, Miaomiao Li

**Affiliations:** 1 Business School of Liaocheng University, Liaocheng, China; 2 University of Wisconsin-Madison, Madison, Wisconsin, United States of America; 3 Business School, Beijing Information Science and Technology University, Beijing, China; Philadelphia University, JORDAN

## Abstract

Based on the panel data of 118 countries from 2013 to 2024, this paper uses the tsQCA method to explore the synergistic effect of various factors of innovation input on innovation output capacity, and reveals multiple paths to improve national innovation output. The results show that there are four ways to improve the output of high innovation, which can be summarized into two models: “Institution + Human Capital and Research + Business Maturity” and “Infrastructure + Market Maturity + Business Maturity”, among which business maturity is an indispensable core factor for countries to improve their innovation output capacity. In the time dimension, the consistency level of the four high-innovation output configuration pathways in 2019–2020 was low. In the spatial dimension, there are obvious regional differences in innovation output among countries. Among them, there is a considerable gap in innovation output capacity in Africa compared with other regions; Influenced by the process of economic transformation and the pace of institutional improvement, the level of innovation output in Eastern Europe lies between that of Europe and America on one hand and Central and South Asia on the other; Central and South Asia has formed a gradient gap with Europe and America as a whole due to insufficient infrastructure and limited market maturity.

## 1 Introduction

Innovation is a prerequisite for the sustainable development of a country and a key factor in enhancing national comprehensive strength and achieving high-quality economic growth [1]. The success of economic development is closely linked to a nation’s ability to acquire, absorb, disseminate, and apply modern technology [2]. Innovation contributes significantly to optimizing industrial structures and improving production efficiency [3], However, [4] it also brings challenges such as high risks associated with research and development investments and exacerbates global competition through increased technological barriers between economies [5]. Strict bilateral and multilateral controls between countries have persisted, making the highly fragmented international division of labor and the closely linked upstream and downstream segments of the global value chain susceptible to disruption risks [6]. The World Intellectual Property Organization’s 2024 Global Innovation Index report (hereinafter referred to as GII) points out that key indicators measuring future innovation activities, such as global venture capital activities and R&D funding, have shown significant weaknesses. The dual constraints of a sluggish global economic recovery and intensifying geopolitical conflicts have become major obstacles to global innovation development [7].

An increasing number of countries recognize that, in addition to breaking technological boundaries and overcoming innovation barriers, unleashing the innovation potential of their citizens is crucial for economic growth and innovation development, as well as for addressing local and global challenges. Research has found that countries that successfully integrate factors influencing innovation exhibit higher innovation outputs and achieve greater economic prosperity [8,9]. The 2024 GII report outlines that innovation inputs encompass five dimensions: institutions, human capital and research, infrastructure, market sophistication, and business sophistication. Governments need to allocate resources reasonably based on their national conditions to promote innovation, leveraging the synergistic effects of government, enterprises, and markets. Therefore, the challenge for policymakers lies in establishing a vibrant innovation ecosystem that enhances innovation output. In other words, a nation’s innovation output depends not only on the critical role of individual factors but also on the synergistic effects resulting from the interactions among these factors [10]. The complex configuration effects among the elements of innovation inputs are key to enhancing a nation’s innovation output.

Due to limitations in research methods and analytical perspectives, previous studies have struggled to deeply analyze the essence and mechanisms of national innovation development, resulting in a lack of clarity regarding the pathways that drive national innovation.

Firstly, most studies follow traditional research methods, employing deductive logic to explore the independent effects of single variables, identifying the positive impacts of various innovation input elements on output. Numerous scholars primarily conduct in-depth analyses of innovation development characteristics within a single country or region, making comparisons between countries or regions [11,12]. Cluster analyses are conducted on countries based on different innovation inputs, outputs, and efficiencies, followed by empirical summarization and qualitative descriptive analysis [13,14]. Through principal component analysis and artificial neural network analysis, the indicators of innovation inputs and outputs are simplified, highlighting the impact of key indicators on output, the relationships between these key indicators, and their effects on GDP and other factors [15,16]. Different support systems and collaborative organizations related to the main industries have formed an interdependent and symbiotic evolving innovation ecosystem [1]. However, the aforementioned studies may overlook other potential influencing factors and the interactions between these factors. It remains unclear whether the insufficiency of individual elements hinders innovation and how the configuration of conditions systematically affects national innovation. Given the varying national contexts and the differing roles of government and market dominance, achieving a “perfect country” with high levels of all innovation input elements is both difficult and unnecessary. Therefore, exploring the combinatory effects among conditions within the innovation input system can not only uncover the configurations that promote innovation output but also explain the mechanisms by which high innovation can be achieved despite the absence of individual elements. Ultimately, this will clarify the more diverse and complex symbiotic, cohabiting, and dominant relationships among the elements within the innovation input system.

Secondly, although some studies have also explored the configurations that influence innovation output, they have employed cross-sectional data to investigate the conditional combinations that lead to high innovation output in countries, comparing the differences in the configurations that generate high innovation between high- and low-income countries [17,18]. However, related research has not thoroughly discussed how specific configurations can consistently generate innovation output. It remains unclear whether the pathways to enhancing innovation output vary over time between different economies. Thus, how can we identify the synergistic effects and interaction mechanisms between innovation input elements and construct diverse pathways for national innovation development? From both temporal and spatial perspectives, how do different countries find the paths most suited to their own innovation development? Addressing these questions from a configurational perspective is of significant importance for enhancing innovation output and achieving national innovation development.

In summary, previous studies have demonstrated that national innovation output is influenced by multiple factors, providing a solid foundation for this paper. However, there is still room for further exploration, particularly in the research on the impact of multi-factor configurations on innovation output, which remains relatively limited. Therefore, using time-series qualitative comparative analysis (tsQCA) to more accurately examine how various innovation input factors affect innovation output is a valuable complement to existing research. The marginal contributions of this paper are as follows: First, in terms of research perspective, previous studies have mostly adopted a single-factor perspective, exploring the linear impact of individual factors on innovation output. However, the influence and driving mechanisms of multiple factors within complex systems on innovation output warrant further investigation. This paper constructs a theoretical analysis framework based on the Global Innovation Index and explores how different combinations of institutional factors enhance innovation output from a configurational perspective, offering a new approach to the analysis of national innovation issues. Second, regarding research methods, most existing studies rely on traditional regression analysis, which requires certain hypothesis testing, and the issue of endogeneity may be difficult to resolve completely. This paper utilizes the tsQCA method to analyze national innovation development issues. This qualitative analysis method, based on causal logic, effectively avoids endogeneity problems, providing accurate empirical evidence on the impact of innovation input factors on innovation output. Third, this paper actively responds to the call from scholars both domestically and internationally for the application of dynamic configurational theory, incorporating the time factor into the analysis of the factors influencing innovation output. Assessing and comparing the impact of innovation input factors on innovation output across countries and over time will contribute positively to advancing dynamic configurational theory research.

## 2 Literature review and theoretical analysis framework

### 2.1 Literature review

National innovation capability positively impacts international competitiveness, enhances productivity, and promotes economic development [19]. The innovation index quantifies national innovation capability, serving to assess what a country should do to encourage innovation and to determine which countries hold advantages relative to others [19]. This study uses GII data because previous innovation indices often selected a few subjectively considered important innovation factors from specific organizations’ data. In contrast, the GII includes data from various organizations and specifically utilizes data from the World Intellectual Property Organization when considering intangible assets. Numerous studies have confirmed that the national innovation capability indicators derived from the GII are highly representative. The GII-2024 tracks both innovation inputs and outputs; the former includes national economic factors capable of conducting innovation activities, while the latter represents the results of innovation activities within the economy. The GII has been widely adopted as a framework for analyzing national innovation, with many studies using the GII dataset to investigate various issues related to national innovation [19–23].

Cavalcante (2022) used the Global Innovation Index as the primary data source and employed cluster analysis to propose an experimental NIS (National Innovation System) type. This type more accurately describes how innovative countries break income constraints to achieve high innovation output. Due to the extensive number of indicators in the GII, some researchers have explored key factors influencing innovation output and the bottleneck factors for high- and low-income countries from the perspective of indicator simplification [4,15]. Bakhtiar et al. (2021) evaluated the heterogeneity of elements within national innovation systems across various dimensions. The study employed two-step clustering techniques and descriptive statistical methods to group countries globally into internally homogeneous and externally heterogeneous groups. Innovation inputs and outputs were classified into high and low levels, providing targeted policy support to different countries to address their innovation output shortcomings [14]. Esteves and Feldmann (2016) also analyzed the correlation between economic variables and the Global Innovation Index. They found that the role of government in providing and creating a supportive institutional environment is crucial for encouraging and supporting innovation. The study revealed significant differences in GII indicators across continents, but found strong homogeneity in innovation inputs and outputs among low-income countries [12]. Choi and Zo (2019) explored the efficiency of innovation in developing countries and indicated that there are significant differences in innovation and operational conditions among these countries.

Although existing research has provided rich explanations for national innovation, it is challenging to offer sufficient theoretical support for the differentiated pathways to enhance national innovation. In reality, improving national innovation output requires countries to adopt different matching approaches for innovation input elements based on their own needs, conditions, and characteristics. The configurations that lead to the emergence of outcome variables, as well as those that cause the disappearance of outcome variables, may differ across countries. To address these limitations, this paper will introduce the tsQCA method under the GII framework to explore the synergistic effects of innovation inputs and reveal the complex relationships among various influencing factors.

### 2.2 Analysis framework

In the 2024 GII report, innovation inputs include five factors: institutions, human capital and research, infrastructure, market sophistication, and business sophistication. These five factors are closely related to innovation output.

Institutions and Innovation Output. In the GII framework, institutions include political systems, regulatory systems, and business environment systems. North’s research found that institutions play a significant role in encouraging innovation in terms of economic incentives. Institutions can affect the level of market activity [24]. A stable political environment can reduce operational uncertainty, serving as a key driver of economic activities and promoting innovation. An improved regulatory system contributes to the enhancement of the national governance system and modernization of governance capabilities, helping to continuously optimize the business environment and promote high-level technological cooperation and exchange. The improvement of business environment institutions can reduce the uncertainty in innovation and entrepreneurship, positively impacting corporate governance, competitive advantages, and innovation capabilities [25].

Human Capital and Research and Innovation Output. In the GII framework, human capital and research include Education, Higher Education, and Research and Development. Studies have found that the level of education and research activities is a major determinant of creative output, with university research having a significant impact on business patents and national innovation [26]. Dakhli and De Clercq (2004) studied the impact of human capital on national innovation using data from the *World Development Report*. The study found that a country’s overall human capital is related to innovation-related skills, such as the number of professionals engaged in R&D activities [27]. The development of human capital can promote innovation, which in turn transforms innovative outcomes into intangible assets for a company or a country [28].

Infrastructure and Innovation Output. In the GII framework, infrastructure includes information and communication technology, general infrastructure, and ecological sustainability. Research has found that infrastructure is a fundamental element of economic development [29], with communication, transportation, and energy infrastructure facilitating the exchange of innovative ideas and services. Malecki emphasizes that changes in infrastructure have an impact on economic growth and development. General infrastructure, as a fundamental element of regional development policy strategies, increases the accessibility of resources and enhances productivity. The improvement in productivity attracts R&D-intensive enterprises, such as research institutions, universities, and corporate research institutes, which in turn fosters the concentration of human capital, particularly researchers [29]. This, in turn, results in increased R&D investment, a higher number of university graduates, greater educational investment, and improved innovation capabilities. Ecological sustainability promotes social welfare, reduces economic costs, and saves fiscal resources [30]. In the digital age, some beliefs, mindsets, and ingrained practices hinder the broader and faster adoption of new online collaboration practices [31]. Building trust, generating rapid feedback, or sharing local environments are key features of synchronous interaction, which are considered to be insufficiently supported by information and communication infrastructure [32].

Market Maturity and Innovation Output. In the GII framework, market maturity includes credit, investment, trade competition, and market size. The credit system ensures the legal rights of both borrowers and lenders, thereby increasing investment in innovation activities [33]. Lumpkin and Dess’s research points out that distinguishing between market innovation and technological innovation can provide a useful measure for conceptualizing innovation. In this regard, market innovation can be considered an important input factor in the innovation process [34]. Research has found that foreign direct investment (FDI) has a significant impact on productivity growth and knowledge diffusion [35]. Additionally, some studies have demonstrated the significant impact of knowledge capital disclosure on market value. Market value is closely related to patent citation indicators, and innovation depends on technological characteristics such as patent applications and market structure [19]. For example, during the pandemic, the Johns Hopkins University Center for Health Security advocated for the creation of a global vaccine platform to integrate all efforts regarding scientific resources for vaccine development. When the pandemic broke out, governments and companies were willing to heavily fund vaccine research. However, as the pandemic waned, investor interest also declined. Vaccines are subject to systemic underinvestment in R&D by private pharmaceutical companies because the societal benefits (social returns) from innovation exceed the returns that innovators receive (private returns) [7].

Business Sophistication and Innovation Output. Within the Global Innovation Index (GII) framework, business sophistication includes knowledge workers, innovation linkages, and knowledge absorption. National innovation capacity is viewed as a developmental process. Todorova and Durisin suggest that knowledge absorption is a continuous feedback loop involving acquisition, assimilation, transformation, and exploitation. Knowledge continuously accumulates, and the absorption of new knowledge is embedded in current operational processes. At the national level, the concentration of knowledge workers leads to increased emphasis and investment in education in a region. Good cooperation experiences between academia, research, and industry can trigger more research institutions to serve enterprises, thereby increasing enterprise R&D investment. The purchase and sale of intellectual property and the higher proportion of research talent will lead governments and enterprises to place greater emphasis on education and research and development [36]. Companies develop productivity, competitiveness, and innovation potential by hiring highly qualified professionals and technical personnel [37]. Therefore, business maturity can enhance innovation outcomes. Hall and Bagchi-Sen studied the relationship between R&D intensity, innovation measures, and company performance. The results showed that R&D intensity is related to patent measures, while innovation is associated with the introduction of new products and the measurement of company performance [38]. Hernan, Marin, and Siotis found that R&D intensity, industry concentration, company size, technology spillovers, and research have a strong influence on the formation of research-oriented joint ventures [39]. Connolly’s research found that the absorption of knowledge through the acquisition of intellectual property and high-tech imports not only determines GDP growth but also influences domestic innovation [40].

**Fig 1:**
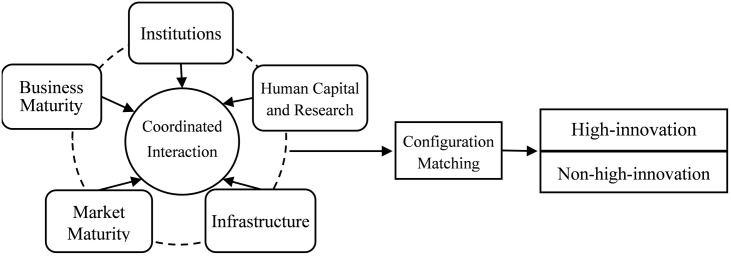
Theoretical model.

In summary, this paper adopts the framework of the GII, constructing a research model that includes five key elements driving the enhancement of national innovation output: institutions, human capital and research, infrastructure, market maturity, and business maturity. Using a configuration perspective and a dynamic QCA method based on panel data, the study analyzes countries included in the GII indicator system as case studies. The research constructs a two-dimensional analytical framework that incorporates temporal and spatial dimensions to explore the multiple paths through which these antecedent conditions, in their combined configurations, impact the improvement of national innovation output. The study summarizes the experiences of high-innovation countries and identifies the shortcomings of non-high-innovation countries, aiming to provide theoretical support and practical reference for countries to enhance innovation output according to their specific characteristics. The theoretical analysis framework is illustrated in Fig 1.

## 3 Research design and data calibration

### 3.1 Research methodology

The traditional QCA method has a theoretical limitation in that it overlooks the time and space dimensions of data. Additionally, due to the limitations of analytical tools, most existing research uses static cross-sectional data to explore the influencing factors at a specific time point, making it difficult to analyze causal relationships that change over time. This approach fails to explore the configurational effects along the time dimension. Since national innovation capacity is a continuous process, the factors affecting national innovation output evolve over time. These are events that occur continuously along the time axis, and relying solely on cross-sectional data cannot explain the interactions between causality and time. To address the continuous time span effects between variables, Hino developed Time-Series Qualitative Comparative Analysis (tsQCA), which analyzes the configurational results and their changes across groups, both within and between groups, as well as overall consistency and coverage. This method effectively resolves the static problem inherent in traditional QCA. Based on this, this study adopts the tsQCA method, focusing on the combinations of multiple factors at different time points. It will better reveal the different paths formed by the combinations of antecedent conditions in national innovation output capacity, and provide a deeper analysis of the complex, nonlinear, and asymmetric causal relationships between antecedent condition variables and national innovation output capacity.

### 3.2 Research sample

Following the basic principle of “sufficient and accessible case sample size,” this study selects 118 countries as research cases, considering that data for some countries is missing for certain years. The data comes from the Global Innovation Index Research from 2013 to 2024.

### 3.3 Calibration

Based on existing theories and previous research, this study performs a unified calibration of the data to facilitate subsequent analysis of within-group, between-group, and overall consistency and coverage. According to the characteristics of the variable values in this study, the direct calibration method is used, setting the 95th percentile, 50th percentile, and 5th percentile as calibration anchors, representing full membership, the crossover point, and full non-membership, respectively. The specific calibration results are shown in Table 1.

**Table 1:** Variable calibration.

Variable Names		Calibration		
		Full Membership	Crossover Point	Fully Non-membership
Result Variables	Innovation Output Y	53.416	27.175	9.900
Causal Variables	Institutions X1	90.025	61.871	37.175
	Human capital and research X2	61.150	32.200	12.400
	Infrastructure X3	63.200	43.300	21.600
	Market Maturity X4	68.791	45.500	23.174
	Business Maturity X5	59.005	31.063	17.400

The calibration process uses RStudio softwareto convert the original data into fuzzy set membership scores between 0 and 1. The core code is as follows (For the complete code, please refer to the data availability statement in this paper):

# Define the calibration threshold of each variable (e = fully non-membership, c = crossover point, i = fully membership)

calibrations <- list(Y = c(e = 9.9, c = 27.175, i = 53.416),

X1 = c(e = 37.175, c = 61.871, i = 90.025),

X2 = c(e = 12.4, c = 32.2, i = 61.15),

X3 = c(e = 21.6, c = 43.3, i = 63.2),

X4 = c(e = 23.174, c = 45.5, i = 68.791),

X5 = c(e = 17.4, c = 31.063, i = 59.005))

# Loop to realize direct calibration of all variables

for (var in names(calibrations)) {jepm[[var]] <- calibrate(jepm[[var]], type = “fuzzy”, method = “direct”, thresholds = calibrations[[var]])}

After the code runs, the obtained csv format file is the calibrated data. Because QCA software can’t recognize the value of variable 0.5, in order to avoid that the fuzzy set membership is exactly 0.5 and can’t be included in the analysis, this study replaces 0.5 with 0.501.

## 4 Results analysis

### 4.1 Necessity analysis of individual conditions

Similar to the traditional QCA method for testing necessary conditions, the criterion for necessity analysis is determined by the level of consistency. When the consistency score is between 0.8 and 0.9, the condition variable can be regarded as a sufficient condition for the outcome variable; when the consistency score exceeds 0.9, the condition variable can be considered a necessary condition for the outcome variable, meaning that the condition is indispensable for the occurrence of the outcome. In tsQCA analysis, Garcia et al. [41] suggest further calculating the consistency adjustment distance to assess the robustness of the necessary condition. When the adjustment distance is less than 0.2, the aggregated consistency accuracy is higher, providing stronger support for the judgment. When the adjustment distance is greater than 0.2, it indicates that the experiment is influenced by time effects and individual effects, requiring further investigation of the condition’s necessity.

According to Table 2, when the outcome variable is high and non-high innovation output, the aggregated consistency of the five condition variables—institutions, human capital and research, infrastructure, market maturity, and business maturity—are all less than 0.9, failing to meet the consistency requirement. This indicates that no single factor constitutes a necessary condition for high or non-high innovation output. However, the within-group consistency adjustment distances are generally greater than 0.2, suggesting the presence of significant regional effects between the five antecedent conditions and the outcome. In the between-group consistency adjustment distances, there are four cases where the distance exceeds 0.2, indicating the need for further analysis of potential time effects.

**Table 2:** Necessity test of a single conditional variable.

Condition Variables	High innovation output				Non-high innovation output			
	Cumulative Consistency	Cumulative Coverage	Between-group consistency adjustment distance	Within-group consistency adjustment distance	Overall consistency	Overall coverage	Between-group consistency adjustment distance	Within-group consistency adjustment distance
Institutions	0.830	0.817	0.144	0.264	0.508	0.511	0.238	0.495
~Institutions	0.503	0.499	0.125	0.528	0.818	0.832	0.093	0.253
Human Capital and Research	0.854	0.855	0.086	0.297	0.480	0.493	0.074	0.561
~ Human Capital and Research	0.494	0.481	0.082	0.550	0.859	0.858	0.062	0.231
Infrastructure	0.843	0.821	0.167	0.275	0.500	0.498	0.164	0.528
~ Infrastructure	0.485	0.486	0.206	0.550	0.820	0.842	0.125	0.242
Market Maturity	0.828	0.808	0.148	0.165	0.550	0.550	0.374	0.407
~ Market Maturity	0.539	0.539	0.125	0.462	0.809	0.828	0.140	0.231
Business Maturity	0.860	0.882	0.055	0.231	0.468	0.491	0.292	0.539
~ Business Maturity	0.504	0.480	0.086	0.517	0.887	0.866	0.043	0.165

Note: ~ denotes logical NOT

Further analysis of the causal relationship combinations with between-group consistency adjustment distances greater than 0.2 is presented in Table 3. In all four cases, the between-group consistency levels are less than 0.9, meaning that none of the conditions, when considered over the entire time period, constitute a necessary condition for increasing national innovation output. This indicates that innovation output is a complex system, and it is necessary to conduct a configuration analysis of these condition variables to identify the various combinations of conditions that influence high or non-high innovation output.

**Table 3:** Causal combination of consistency adjustment distance between groups greater than 0.2.

Situation	Causal Combination	Indicator	Year											
			2013	2014	2015	2016	2017	2018	2019	2020	2021	2022	2023	2024
Situation 1	X1/ ~ Y	Between-group consistency	0.626	0.605	0.577	0.545	0.532	0.544	0.585	0.57	0.586	0.427	0.314	0.277
		Between-group coverage	0.430	0.459	0.456	0.490	0.502	0.515	0.552	0.588	0.578	0.582	0.485	0.476
Situation 2	~X3/Y	Between-group consistency	0.679	0.611	0.542	0.470	0.401	0.424	0.400	0.446	0.464	0.383	0.410	0.459
		Between-group coverage	0.653	0.616	0.595	0.530	0.518	0.49	0.474	0.363	0.405	0.343	0.371	0.388
Situation 3	X4/ ~ Y	Between-group consistency	0.739	0.802	0.750	0.592	0.632	0.648	0.666	0.621	0.600	0.250	0.273	0.253
		Between-group coverage	0.466	0.519	0.523	0.563	0.564	0.581	0.597	0.640	0.610	0.528	0.465	0.467
Situation 4	X5/ ~ Y	Between-group consistency	0.642	0.603	0.687	0.563	0.569	0.496	0.503	0.336	0.317	0.372	0.365	0.354
		Between-group coverage	0.452	0.481	0.508	0.529	0.530	0.507	0.526	0.485	0.457	0.499	0.437	0.465

### 4.2 Sufficiency analysis of condition configurations

The core of the QCA method is the configuration analysis, which aims to examine how different combinations of antecedent conditions influence the outcome. The threshold of the number of cases selected in this study is 1, the original consistency threshold is 0.75, and the PRI threshold is 0.6, which finally covers 1367 cases. Following Park et al.’s research, the conditions that appear in both intermediate and simple solutions are defined as core conditions, conditions that appear in the intermediate solution but not in the simple solution are defined as auxiliary conditions, and conditions that do not appear in either the simple or intermediate solutions are regarded as conditions that can either exist or not. The results are shown in Table 4.

**Table 4:** Configuration results of high and non-high innovative outputs.

Condition Variables	High-innovation output configuration				Non-high-innovation output configuration					
	M1		M2							
	Configuration a1	Configuration a2	Configuration a3	Configuration a4	Configuration b1	Configuration b2	Configuration b3	Configuration b4	Configuration b5	Configuration b6
Institutions	●	●	●			ς	ς	ς		
Human Capital and Research	●	●		●	ς	ς	ς		ς	
Infrastructure	●		●	●		Υ		Υ	ς	Υ
Market Maturity		●	●	●			Υ		ς	ς
Business Maturity	●	●	●	●	ς			ς		ς
Consistency	0.959	0.968	0.962	0.968	0.925	0.931	0.95	0.938	0.948	0.941
PRI	0.929	0.943	0.931	0.943	0.859	0.867	0.896	0.878	0.895	0.772
Coverage	0.682	0.654	0.651	0.662	0.796	0.698	0.667	0.699	0.662	0.418
Unique coverage	0.051	0.023	0.019	0.030	0.048	0.013	0.005	0.022	0.004	0.018
Between-group consistency adjustment distance	0.035	0.023	0.031	0.027	0.074	0.078	0.047	0.066	0.066	0.039
Within-group consistency adjustment distance	0.039	0.035	0.043	0.035	0.043	0.043	0.043	0.066	0.066	0.039
Overall consistency	0.959				0.925					
Overall PRI	0.929				0.859					
Overall Coverage	0.682				0.796					

Note: ●** =** The core condition exists; ς = The core condition is missing; ● = The auxiliary condition exists; Υ = The auxiliary condition is missing; A blank indicates that the condition does not affect the outcome.

#### 4.2.1 Summary of results.

Schneider and Wagemann proposed that the consistency of a single solution and the overall solution should not be lower than 0.75. According to Table 4, the overall consistency of high and non-high innovation output solutions are 0.959 and 0.952, indicating that the configurations can be regarded as sufficient conditions for high (non-high) innovation output. The overall coverage is 0.682 and 0.796, respectively, suggesting that the configurations explain a high proportion of high (non-high) innovation output. High innovation output has four configuration paths, which can be divided into two types: M1, composed of configuration a1 and a2, is named as “Institution + Human Capital and Research + Business Maturity” driven type; M2, composed of configuration a3 and a4, is named as “Infrastructure + Market Maturity + Business Maturity” driven type. Non-high innovation output has six configuration paths. This paper focuses on the theoretical implications of high innovation output configurations and analyzes corresponding cases. The configuration path of high innovation output is summarized in Table 5.

**Table 5:** Summary of configuration path of high innovation output.

Innovation output-driven type	Configuration	Core Condition	Auxiliary Condition	Typical Country	Consistency	Coverage
M1: “Institution + Human Capital and Research + Business Maturity” Driven Type	Configuration a1	Institution; Human Capital and Research; Business Maturity	Infrastructure	Britain, France	0.959	0.682
	Configuration a2		Market Maturity	The United States	0.968	0.654
M2: “Infrastructure + Market Maturity + Business Maturity” Driven Type.	Configuration a3	Infrastructure; Market Maturity; Business Maturity	Institution	Japan	0.962	0.651
	Configuration a4		Human Capital and Research	China	0.968	0.662

M1: “Institution + Human Capital and Research + Business Maturity” Driven Type. This model mainly includes configurations a1 and a2, both of which have high consistency and coverage, explaining 68.2% and 65.4% of the sample cases, respectively. In this model, institution, human capital and research, and business maturity are common core conditions, while auxiliary conditions exhibit a substitute relationship (infrastructure in a1 and market maturity in a2). Therefore, these two configurations form a second-order equivalent configuration. Configuration a1 is mainly represented by the United Kingdom and France. The United Kingdom has a developed financial system and capital markets, a long-standing research tradition, and a high-quality education system, all of which provide a solid foundation for innovative activities. France, with its rich cultural history and strong education system, demonstrates unique innovation advantages in the humanities and social sciences, while its luxury goods industry enjoys global prestige. The typical country for configuration a2 is the United States. The U.S. government has implemented policies favorable to innovation, such as tax incentives and R&D subsidies, creating a favorable environment for innovation. Additionally, the U.S. is home to numerous world-class higher education and research institutions, which continuously cultivate talent with innovative capabilities and spirit, providing a constant source of motivation for innovation. Moreover, the U.S. has a mature and open business environment with intense competition among enterprises, prompting businesses to continuously innovate to maintain their competitiveness.

M2: “Infrastructure + Market Maturity + Business Maturity” Driven Type. This model mainly includes configurations a3 and a4, both of which have high consistency and coverage, explaining 65.1% and 66.2% of the sample cases, respectively. In this model, infrastructure, market maturity, and business maturity are common core conditions. The difference lies in that configuration a3 emphasizes the level of institutional development, while configuration a4 focuses on human capital and research. Configuration a3 is typically represented by Japan. Japan has well-developed infrastructure and an advanced transportation network, providing convenience for corporate innovation activities. Additionally, Japan’s market and business environment are highly mature, with consumers being highly receptive to new products and technologies, and intense competition among businesses, which drives continuous innovation. Configuration a4 is represented by China. In recent years, the Chinese government has made significant investments in infrastructure construction, including transportation, communications and other fields, providing the necessary material conditions for innovation activities. As the market continues to mature and expand, Chinese companies face increasingly intense market competition, which stimulates their innovative energy. At the same time, China’s business environment is gradually improving, with the government introducing a series of policies to encourage innovation and entrepreneurship, such as simplifying approval processes and enhancing intellectual property protection. These factors work together to drive the rapid growth of innovation output in China.

It is important to note that business maturity is a core condition for generating high innovation output in all four configurations. This indicates that the maturity of the business environment is crucial for stimulating and sustaining high levels of national innovation output. Insufficient business maturity may become a bottleneck restricting the improvement of national innovation output. Therefore, to enhance national innovation output, countries should focus on improving business maturity. This can be achieved through policy formulation, financial investment, international cooperation, and other measures to optimize the market environment, strengthen risk management, and comprehensively enhance business maturity, thereby promoting innovation development.

#### 4.2.2 Inter-group results.

**Fig 2:**
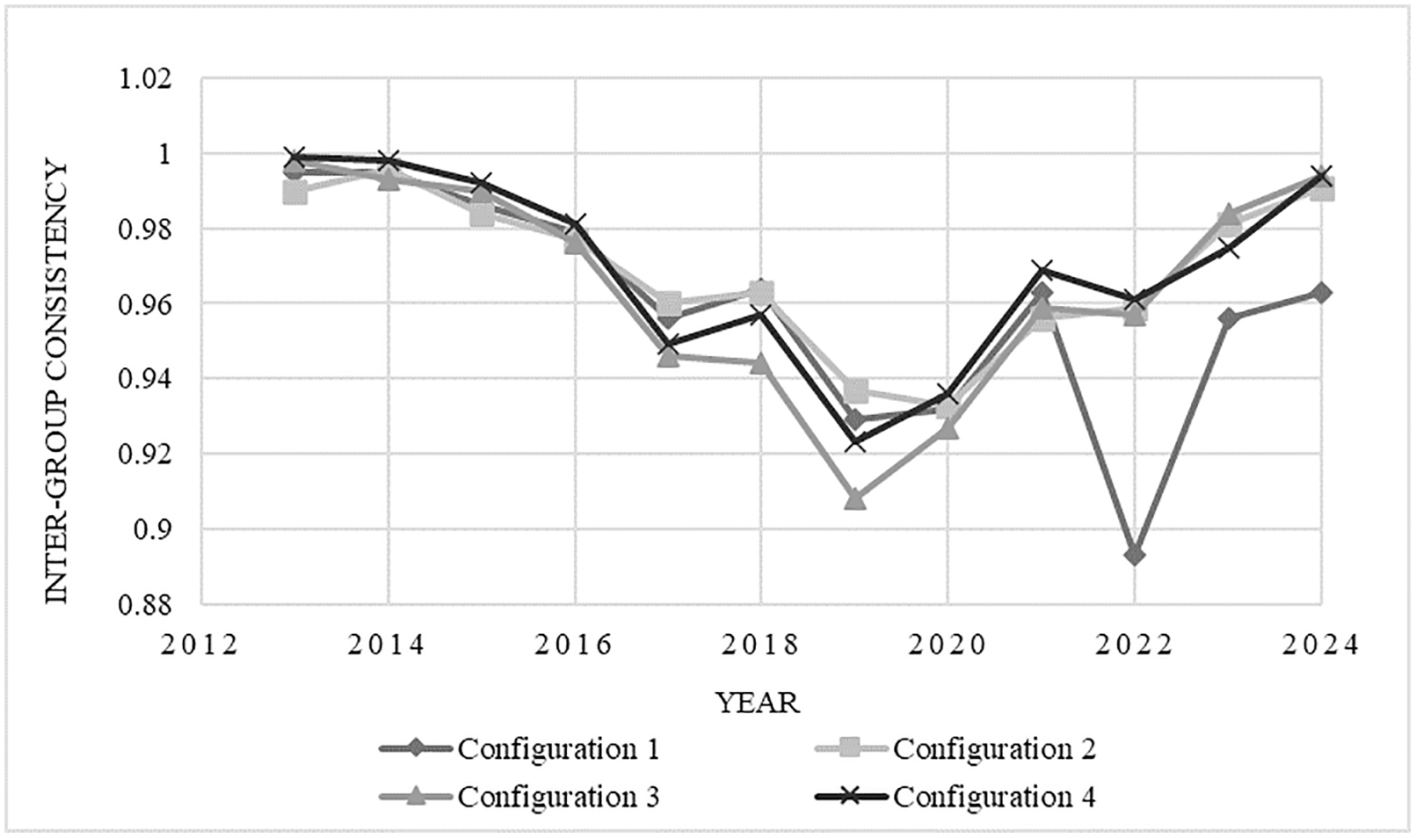
Change of consistency level between groups.

To address the issue of traditional QCA ignoring the time dimension, the inter-group consistency was used to explore the time effects of configurations. The trend of inter-group consistency is shown in Fig 2. Although the inter-group consistency of the four configurations that lead to high innovation output is greater than 0.85, exceeding the consistency threshold of 0.75, and the inter-group consistency adjustment distances are all less than 0.2 (see Table 4), indicating no significant time effects, it is still possible to further examine the changes in each configuration over time through inter-group consistency. From the Fig 2, it can be observed that the consistency levels of all configurations from 2013 to 2024 fluctuate between 0.89 and 1.00, generally stabilizing with strong explanatory power. Notably, the consistency levels in 2019−2020 were relatively lower, and the underlying reason may be the impact of the sudden outbreak of COVID-19. During this period, government attention was primarily focused on epidemic control, and the involvement of enterprises, universities, and research institutions was relatively low, resulting in temporary challenges to the innovation environment. Regional innovation environments faced a temporary shock, especially as domestic and international markets were under significant pressure, leading to low demand. Consequently, the role of various antecedent conditions in improving innovation output was less significant than in previous years. However, this phenomenon does not undermine the explanatory power of configurations regarding the overall situation, and it still provides valuable reference for assessing normal innovation output capabilities. It can be concluded that the existence of the four condition configurations can fully lead to the result, namely, achieving high innovation output.

#### 4.2.3 Intra-group results.

In the data for high innovation output, the intra-group consistency adjustment distances for all four configurations are less than 0.2, indicating that the explanatory power of each configuration does not vary significantly across countries. A statistical analysis of the valid cases for each configuration reveals that the high innovation output configurations can generally be divided into five regions: Europe, America, and Oceania (primarily the United States, Britain, and Australia); Asia and South America (primarily China, Japan, and Brazil); Eastern Europe (primarily Poland and Hungary); Central and South Asia (primarily Kazakhstan, Kyrgyzstan, and Pakistan); and Africa (primarily South Africa, Arab nations, and Egypt).

From the average regional coverage scores in Table 6, it can be observed that the four configurations have the highest coverage in the Europe, and the lowest coverage in Africa.

**Table 6:** Regional coverage average.

Region	M1: “Institution + Human Capital and Research + Business Maturity” driven model		M2: “Infrastructure + Market Maturity + Business Maturity” driven model	
	Configuration a1	Configuration a2	Configuration a3	Configuration a4
Europe, America, and Oceania	0.727	0.682	0.675	0.673
Asia and South America	0.532	0.549	0.556	0.572
Eastern Europe	0.559	0.539	0.510	0.526
Central and South Asia	0.463	0.513	0.499	0.485
Africa	0.338	0.369	0.402	0.327

Specifically, the overall economic development level in Europe, the United States, and Oceania is higher than that in Asia and South America. In Europe, the United States, and Oceania, configurations a1 and a2 have relatively high coverage, with high innovation output driven by well-established institutions, high levels of human capital investment, and mature commercial environments. Typical examples include the United States, the United Kingdom, France, Iceland, and Australia.

In Asia and South America, configurations a3 and a4 have relatively higher coverage. Compared to Europe, the United States, and Oceania, these regions have advantages in infrastructure and market maturity. China, often referred to as the “infrastructure giant,” is a typical example, with its high-speed rail, highways, power grids, and 4G network infrastructure leading the world. In addition, countries like South Korea, Japan, and Thailand, which have relatively weaker institutions and human capital development, still benefit from large markets and intense competition among enterprises, making the “Infrastructure + Market Maturity + Business Maturity” driven path suitable for enhancing innovation output.

The advantages of Eastern European countries in human capital and research foundations are more readily translated through the M1 pathway, yet their overall coverage remains lower than that of Europe, America, and Oceania due to constraints in business sophistication. For instance, Poland has achieved upper-middle levels in education investment and the number of research institutions, and its institutional framework has gradually aligned with international standards since joining the European Union. However, its business environment suffers from inefficiencies in institutional implementation and disruptions in the technology commercialization chain. Countries such as Slovakia and Belarus have inherited solid research foundations from the Soviet era and possess ample human capital, yet they face challenges such as institutional misalignment during market-oriented reforms and underdeveloped business support systems. These issues have collectively limited the effective realization of the M1 pathway’s potential.

The configurational coverage under the M2 pathway in South and Central Asia is relatively higher, which aligns with the reality that some countries in the region rely on resource revenues to advance infrastructure construction. However, shortcomings in market and business sophistication limit the effectiveness of this pathway. For example, Kyrgyzstan has used earnings from energy exports to make certain investments in transport and energy infrastructure, yet it suffers from a small domestic market and weak financial support for innovative enterprises. Pakistan has increased investment in transport and energy infrastructure in recent years through the China-Pakistan Economic Corridor, leading to localized innovation clusters in some cities. Nonetheless, the country faces constraints such as a limited domestic market, weak innovation capacity among SMEs, and insufficient supply of business services. As a result, the M2 pathway only functions effectively in certain sectors, and overall innovation output improves slowly.

In Africa, configuration a3 has the highest coverage. These regions have relatively poor economic foundations, but they have dense populations. They can leverage the demographic dividend, expand the market, open up business space, and convert demographic resources into financial strength. By improving infrastructure, they can narrow the gap with high-level countries and follow the “Infrastructure + Market Maturity + Business Maturity” path to drive high innovation output.

### 4.3 Robustness test

This study conducts a robustness test on the antecedent configurations that lead to high innovation output by increasing the case threshold (from 1 to 2) and the consistency threshold (from 0.75 to 0.8). Ultimately, the resulting configurations are consistent with the original ones, suggesting that this study has strong robustness.

## 5 Conclusion and implications

### 5.1 Research conclusion

This study uses the tsQCA method to explore the synergistic effects of various innovation input factors on innovation output capabilities in 118 countries. By analyzing panel data on national innovation output from 2013 to 2024, the study reveals multiple pathways to improve national innovation output. The findings are as follows: (1) Single innovation input factors do not constitute necessary conditions for improving innovation output. (2) The pathways to high innovation output ability are summarized into two types: “Institution + Human Capital and Research + Business Maturity” driven model and “Infrastructure + Market Maturity + Business Maturity” driven model. Both paths include business maturity, indicating it is an essential core factor for improving innovation output capacity. (3) The inter-group consistency adjustment distance of the configurations is smaller than the judgment standard, showing no significant time effect. However, the configuration solution in certain years is clearly affected by unobserved factors, leading to concentrated changes. (4) The intra-group consistency adjustment distance is also smaller than the judgment standard, showing no significant spatial effect. However, a comparison of typical cases and intra-group coverage reveals significant regional differences in innovation output across countries: Compared with other countries, there is a considerable gap in innovation output capacity in Africa; Due to the unsynchronized pace of economic transformation and system improvement in eastern Europe, the innovation output is in the “middle gradient” and is restricted by the adaptability of business ecology; Central and South Asia has a “low gradient lock-in” in innovation output due to insufficient infrastructure systematization and low market and business maturity. The gap with Africa is mainly reflected in local infrastructure investment, but its core is all restricted by business maturity.

### 5.2 Policy implications

Firstly, for nations pursuing the “Institutional + Human Capital and Research + Business Maturity” innovation pathway, governments must prioritize robust institutional frameworks encompassing intellectual property rights, research ethics governance, and innovation commercialization mechanisms. Strategic human capital investments—spanning vocational training, STEM education, and R&D workforce development—are critical, as exemplified by U.S. legislative initiatives anchoring talent cultivation in national innovation strategies. Multidimensional educational investments should foster interdisciplinary competencies and practical innovation skills, while policy incentives must catalyze industry-academia-research collaboration to accelerate knowledge spillovers and technology diffusion. Market environment optimization requires reducing entry barriers and aligning regulatory frameworks with market-oriented mechanisms to enhance innovation commercialization efficiency. Complementary international cooperation platforms can amplify domestic capabilities through cross-border knowledge exchange, as demonstrated by multilateral R&D partnerships in advanced economies. These integrated interventions collectively establish the legal, human resource, and market infrastructure necessary for sustainable innovation ecosystems.

Secondly, for nations pursuing the “Infrastructure + Market Maturity + Business Maturity” path, governments should prioritize high-quality digital infrastructure like 5G networks and cloud computing. This enhances socioeconomic efficiency while enabling industrial digital transformation, as demonstrated by China’s strategic investments in transportation networks and ICT systems. Such infrastructure expansion facilitates talent, capital, and technology flows – exemplified by China’s high-speed rail network stimulating regional innovation clusters. Concurrently, governments must cultivate enterprise-driven innovation through market-friendly policies. Key measures include incentivizing R&D investments (particularly for SMEs), ensuring fair competition, and strengthening IP protection. China’s reformed market access and enhanced IP regime illustrate effective innovation ecosystem development. Japan’s model of industry-academia-research integration through public-private partnerships demonstrates efficient knowledge commercialization, accelerating technology-to-productivity conversion. Both approaches emphasize coordinated resource mobilization across stakeholders to optimize innovation outcomes.

Thirdly, business maturity is essential for innovation processes and high-output innovation, necessitating targeted strategies by region. For advanced economies in Europe, North America, and Oceania, enhancing business maturity hinges on sustaining technological innovation and optimization: leveraging strengths in advanced technologies and human capital, they should drive the development and application of cutting-edge tools like AI and big data to boost enterprise competitiveness and efficiency, while governments focus on fostering innovation-friendly environments and market access to ignite entrepreneurial vitality. In contrast, mid-developing countries in Asia and South America face dual challenges: they must advance industrial upgrading from labor- to capital/technology-intensive sectors and enhance human capital through talent development, education reforms, and stronger industry-academia-research collaboration. Simultaneously, deepening economic openness and improving the business climate can attract foreign investment and technology, critical for bridging innovation gaps. For eastern European countries, we should focus on the collaborative optimization of “institution-business ecology-human capital”. On the one hand, accelerate the reform of system implementation, such as simplifying the administrative examination and approval process of innovative enterprises, improving the enforcement rules of intellectual property protection, and narrowing the gap between system implementation and European and American countries. On the other hand, we can rely on the innovation cooperation platform within the EU to attract European and American enterprises to settle in, and enhance the local business maturity through “bringing innovation with business”. For Central and South Asia countries, priority should be given to the two-wheel drive of “infrastructure systematization +commercial maturity cultivation”. Expand the domestic demand market, improve the financial support system, such as setting up policy banks for innovative small and medium-sized enterprises, and reduce the cost of innovative financing for enterprises. In addition, we can combine our own resource advantages to develop characteristic innovation industries, such as new energy technology and deep processing technology of agricultural products, promote the maturity of market and business environment with “characteristic industry innovation” and gradually break the “low gradient lock-in” of innovation output. For African nations with fragile economic bases, priorities include foundational steps: strengthening infrastructure and investment environments to attract foreign capital and aid, implementing SME-friendly policies to stimulate employment and revenue, and engaging in international cooperation to absorb global best practices, thereby injecting momentum into sustainable economic and innovative growth.

### 5.3 Limitations and outlook

This study also has the following limitations that need to be addressed in future research. Firstly, the research variables were selected based on previous studies, using data from the Global Innovation Index (GII). However, the study only focused on countries with complete data from 2013 to 2024, and some countries had missing data for certain years. The study did not analyze countries with missing data, so future research could include these countries for a more comprehensive analysis. Secondly, based on the GII data framework, this study only analyzed the impact of five innovation input factors: institution, human capital and research, infrastructure, market maturity, and commercial maturity. However, it did not cover all possible factors that could influence innovation. Related research also indicates that cultural and social psychological factors can impact innovation output [42,43], which may result in an incomplete assessment of the innovation potential of certain countries or regions. Therefore, future research could further refine the analytical framework.
