# Correction: Research on multiple improvement paths of national innovation output based on tsQCA

**DOI:** 10.1371/journal.pone.0345673

**Published:** 2026-03-24

**Authors:** The PLOS One Staff

Table 4 was uploaded incorrectly. Please see the correct Table 4 here.

The publisher apologizes for the error.

**Table 4:** Configuration results of high and non-high innovative outputs.

Condition Variables	High-innovation output configuration				Non-high-innovation output configuration					
	M1		M2							
	Configuration a1	Configuration a2	Configuration a3	Configuration a4	Configuration b1	Configuration b2	Configuration b3	Configuration b4	Configuration b5	Configuration b6
Institutions	●	●	●			ς	ς	ς		
Human Capital and Research	●	●		●	ς	ς	ς		ς	
Infrastructure	●		●	●		Υ		Υ	ς	Υ
Market Maturity		●	●	●			Υ		ς	ς
Business Maturity	●	●	●	●	ς			ς		ς
Consistency	0.959	0.968	0.962	0.968	0.925	0.931	0.95	0.938	0.948	0.941
PRI	0.929	0.943	0.931	0.943	0.859	0.867	0.896	0.878	0.895	0.772
Coverage	0.682	0.654	0.651	0.662	0.796	0.698	0.667	0.699	0.662	0.418
Unique coverage	0.051	0.023	0.019	0.030	0.048	0.013	0.005	0.022	0.004	0.018
Between-group consistency adjustment distance	0.035	0.023	0.031	0.027	0.074	0.078	0.047	0.066	0.066	0.039
Within-group consistency adjustment distance	0.039	0.035	0.043	0.035	0.043	0.043	0.043	0.066	0.066	0.039
Overall consistency	0.959				0.925					
Overall PRI	0.929				0.859					
Overall Coverage	0.682				0.796					

Note: ● = The core condition exists; ς = The core condition is missing; ● = The auxiliary condition exists; Υ = The auxiliary condition is missing; A blank indicates that the condition does not affect the outcome.
